# Exploring student perceptions of ward simulation as an exercise to improve decision-making skills in the clinical context

**DOI:** 10.15694/mep.2018.0000018.1

**Published:** 2018-01-18

**Authors:** Calum MacMillan, George Hogg

**Affiliations:** 1 University of Dundee

**Keywords:** Simulation, Decision-making, Non-technical skills

## Abstract

This article was migrated. The article was marked as recommended.

**Introduction:** At the point of graduation, medical students are expected to demonstrate competence making a range of decisions in the clinical environment. However, research continues to highlight that both students and graduates lack effective methods for dealing with complex decisions, with students emphasising having little, if any, experience of making many clinical decisions during their undergraduate training. To combat this, an Acute Medical Unit ward simulation exercise (AMUWSE) has been recently incorporated into the 4 ^th^ year curriculum at Dundee Medical School to aid the development of these skills.

**Methodology:** This study aimed to explore how students perceive their decision-making ability is influenced by an AMUWSE and how this teaching exercise could be improved. An action research approach was adopted to investigate medical students’ perceptions of how participation in this exercise influences clinical decision-making skills. An initial online questionnaire was circulated before four group interviews were conducted.

**Results:** It was found that the majority of students perceived the AMUWSE to have been of significant benefit in developing clinical decision-making. A number of learning points from the exercise were identified, incorporated into six overarching themes: *making clinical decisions, prioritising, handing over information, asking for help, dealing with time constraints* and *preparation for clinical practice.* Within many of these themes, students expressed deficiencies in teaching during the “basic science” years of medical school.

**Conclusions:** This study highlighted the effectiveness of an AMUWSE to improve the clinical decision-making ability of medical students, while offering invaluable insights into the nature of clinical practice. Despite this, areas for improvement have been identified to make this simulation exercise a more effective means of improving clinical decision-making. Furthermore, there remains a need for consideration of how these skills can be developed during the early years of medical school. While a complex process, certain teaching interventions, such as an AMUWSE, have shown potential in aiding this development; helping to create more competent, well-equipped junior doctors.

## Introduction

Over the last decade the need for greater appreciation of the complex nature of patient safety has become increasingly apparent, with acknowledgment that adverse events are commonly caused by more than mere technical failings ( Flin et al., 2008). Adverse events can be classified into many subsets, with preventable medical error forming a key component ( Leape et al., 1991). In most instances, these errors can be attributed to ineffective non-technical skill behaviours, such as recognition of deteriorating patients or inaccurate diagnostic decision-making ( Hogan et al., 2012). The non-technical skills (NTS) identified by Mellanby et al. (2013) as being essential for newly qualified doctors to demonstrate include clinical decision-making, situational awareness, task management and inter-professional teamwork.

With regards to the skill of clinical decision-making specifically, this can be defined as:

*“..a contextual, continuous, and evolving process, where data are gathered, interpreted, and evaluated in order to select an evidence-based choice of action.” (* Tiffen et al., 2014: p.401)

It is critical that medical students, at the point of graduation, display competence making a range of clinical decisions ( General Medical Council, 2015). Despite these expectations, students continue to lack effective methods for dealing with complex decisions and prioritising responsibilities. Students also stress that they have little, if any, experience of making a number of clinical decisions during medical school that they will be expected to make upon graduation ( McGregor et al., 2012). Therefore, there is an apparent need for improved teaching on this subject within undergraduate medical education.

The majority of literature on teaching clinical decision-making has found there tends to be a focus on making a diagnosis, with other aspects such as prioritisation, recognising limits and asking for help often neglected ( Lake, 2005). It is therefore unsurprising that evidence capturing the perceptions of junior doctors has found that graduates are least prepared in basic ‘doctoring skills’, with substandard clinical decision-making a consistent finding ( Wall et al., 2006). Additionally, Illing et al. (2013) inform that graduates feel particularly unprepared for areas of practice based on experiential learning, such as making clinical decisions and managing patients. In order to develop these skills, students require the opportunity to perform ‘on-the-job learning’, through participation in simulated and ward-based experiences.

At the University of Dundee, an Acute Medical Unit ward simulation exercise (AMUWSE) has been recently incorporated into the fourth-year curriculum. This was piloted in September 2016, offering attendees the opportunity to spend a half-day in the Dow Clinical Simulation Suite assuming the role of a ward-round team member. Students were posed the challenges of clerking admissions, managing patients and presenting cases; offering them the opportunity to practice their procedural, examination and non-technical skills.

## Methodology

This study was a continuation of an action research cycle previously initiated, aiming to gather and analyse data from participants to produce recommendations on the teaching of clinical decision-making. Action research is conducted with the intention of implementing positive change and improving practices, through sequential cycles of identifying an issue, planning a change, implementing and observing said change before reflecting on the findings of this process ( Kemmis & McTaggart, 2005; Gray, 2014). In this study, the issue identified was anecdotal evidence of a lack of effective NTS teaching in the Transition Block of the Dundee medical curriculum. The change implemented was the introduction of a new Acute Medical Unit ward simulation exercise for fourth-year students and through exploration of their perceptions, the impact of this change could be ascertained.

Participants were recruited via email, which was distributed to 168 fourth-year students within Dundee Medical School as they were the only population to have undertaken the ward simulation exercise being investigated. The initial email contained information regarding the basis of the research, including a link to the online questionnaire. The only inclusion criteria for this study was students must have had completed the AMUWSE prior to participating.

The questionnaire consisted of nine questions, focused around key decision-making elements ( Table 1) extracted from the FoNTS taxonomy ( Mellanby et al., 2013). A Likert scale approach was adopted - ranging from one ( *‘Strongly Disgaree’*) to five ( *‘Strongly Agree’*) - for the initial seven questions. This was followed by an open question, offering students the opportunity to describe clinical decisions they have made following the exercise and whether they believe they could have made these decisions prior to the AMUWSE, as a result of the AMUWSE or as a result of ensuing clinical experience during fourth-year placements.

Following this, four group interviews were conducted to explore the findings of the questionnaire in greater detail. Each group contained three to five members in each of the four interviews conducted, interviewing 17 individuals in total. As many students were away from Dundee on out-block and clinical placements during the time of these interviews, it limited access for a large sample to be obtained. These were performed until data saturation was reached. Through collecting two different sources of data and completing a review of the relevant literature, triangulation of data was achieved.

Regarding data analysis, all quantitative data was analysed using IBM SPSS software. Qualitative data collected was transcribed verbatim and analysed using Braun and Clarke’s (2006) six-phase guide to thematic analysis, to reveal overarching themes. This was performed inductively, with themes and sub-themes identified peer reviewed to add credibility to the results.

## Results

### Questionnaire

The online questionnaire was sent to all fourth-year students and only those who had completed the AMUWSE were invited to participate. In total, 13 responses were received, a response rate of 7.7%. While lower than the researcher had hoped, this was still sufficient to elicit trends in the scoring statements devised.

The questionnaire firstly explored a range of decision-making elements before offering participants the opportunity to describe clinical decisions they have made since completing the AMUWSE. Students were asked to reflect and express if they feel they could have made these decisions:

• Prior to the AMUWSE
• As a result of the AMUWSE
• As a result of clinical experience during 4 ^th^ year placements

This demonstrated that while many respondents feel clinical experience has been the greatest asset to developing decision-making ability, 23.1% perceive the ward simulation experience to have been of significant benefit.

**Figure 1.**
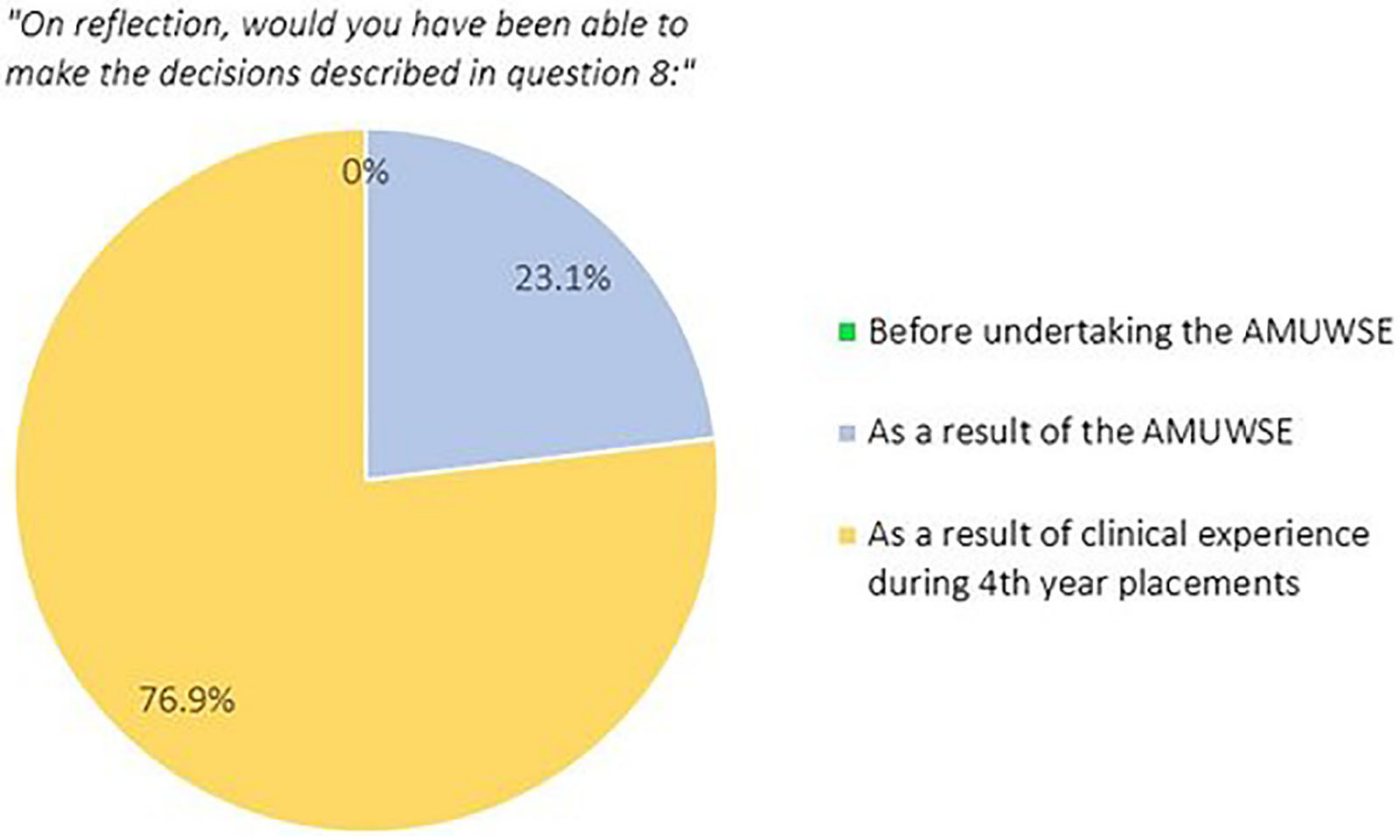
Perceptions of most effective decision-making learning environment

Table 1 showcases the perceptions of students regarding the AMUWSE and its ability to improve several clinical decision-making elements. From this, it appears that the AMUWSE has been beneficial to participants decision-making ability. In response to six of the seven statements, either *‘Agree’* or *‘Strongly Agree’* were selected on between 69.2% and 92.3% of occasions. The only statement receiving a less favoured response was the ability to “ *identify risks and benefits of potential action plans*”.

**Table 1.** Perceptions of the AMUWSE’s influence on decision-making skills

*The AMUWSE improved my ability to:*	Mean
***1. Verbalise or document differential diagnoses:***	3.69
***2. Work with colleagues to help generate differential diagnoses:***	3.77
***3. Identify when senior/more qualified help is required:***	4.15
***4. Appreciate my own skills when choosing an action plan:***	4.08
***5. Appreciate my own limitations when choosing an action plan:***	3.92
***6. Identify risks and benefits of potential action plans:***	3.38
***7. Assess time constraints when considering courses of action:***	3.69

#### Group Interviews

A number of learning points from the exercise were identified following analysis of the interview transcripts. These were incorporated into six overarching themes: *making clinical decisions, prioritising, handing over information, asking for help, dealing with time constraints* and *preparation for clinical practice.* These themes contained several sub-themes and within many of these, students expressed deficiencies in teaching during the initial years of medical school. Each quote discussed in this section is labelled, relevant to their originating group and participant. For instance:

(2.3.) = Group 2, Participant 3

#### Making clinical decisions

All students described that during the AMUWSE they were presented with the reality of making clinical decisions and dealing with the outcomes. Making active decisions proved to be very different in practice than in theory. With the first three years of teaching at Dundee Medical School being largely theory-based, students found the experience to actively make these decisions advantageous.

*“..before the exercise you’d just say, ‘I want to order bloods’..but in the AMUWSE you think ‘okay, how actually do I do that?’”* (2.4.)

Participants could also appreciate how frequently protocols are employed when managing patients, and how these drive clinical decision-making. Despite relieving the responsibility associated with making decisions, students believed protocols helped build their confidence in this skill.

*“..[my case] was a query sepsis, so you don’t really make the decisions yourself, you just follow the protocol”* (2.2.)

*“..for us students who have so little experience, protocol is good enough for us”* (3.2.)

#### Prioritising

Many students recognised the need to prioritise which patients required their immediate help, which often came as a novel experience.

*“..although your patients are important, the ones that you can help right now you should..it made you think ‘what do I have to do that is going be best here and now?’”* (1.1.)

There was also a need for undergraduates to prioritise which patients required greater support than others, placing responsibility on students to make these decisions. While many students appreciated the need to prioritise their patients, a few also acknowledged the importance of this skill when it came to deciding on investigations.

*“I think it helps to weigh up what tests need to go first..what can wait and what can’t wait”* (1.5.)

However, it was perceived that such prioritisation strategies had never been formally taught prior to the AMUWSE, with this exercise offering new and valuable insights.

*“I don’t remember ever having any specific teaching on how to prioritise tasks”* (4.5.)

#### Handing over information

All students were grateful for the opportunity to present patient cases to their seniors, learning what information was essential to include. This experience was relatable to tasks required in the clinical environment, allowing many students to articulate the value in the teaching.

*“..you can relate it to life on the ward if you’re asked to go and see a patient and then report back to your consultant or registrar”* (1.1.)

When handing over information to seniors, students found they often included unnecessary details. It was acknowledged that these are often challenging encounters, requiring quick decision-making. It was stressed by these students that this was a skill that had lacked dedicated teaching, with the AMUWSE presenting an opportunity for development.

*“I felt a bit out of my depth when I was presenting because I did it totally wrong, just because I’d never been taught formally how you should present”* (4.5.)

#### Asking for help

During the exercise, students felt comfortable asking for help when they required it, appreciating at times they were out of their depth. All students also discussed approaching the consultant present on the ward to gain support when making decisions or forming differentials. It was described how discussions with consultants offered important insights for future improvement.

*“..it was good to have an actual consultant on the ward who you can ask ‘have I done this right?’, so you can know for future”* (3.3.)

*“..it made me realise that it is okay to ask for help, that we are out of our depths”* (4.3.)

When asking for help, students discussed approaching their peers for support when making decisions, with many doing this before escalating to a consultant. Peers would often offer reassurance, helping participants to develop their confidence.

*“..in real-life you wouldn’t go up to the consultant straight away and ask them, you would probably speak to your immediate colleagues first”* (1.3.)

*“I think it showed how important it is to speak to a colleague”* (2.3.)

#### Dealing with time constraints

Many students valued the time pressured nature of the exercise, requiring them to make fast decisions regarding their patient case.

*“I think it is more accurate of assessing your own ability if it’s in a [time] pressured situation like that”* (4.4.)

Due to the time constraints on the exercise, students stated they did not complete all tasks required of them, due to substandard decision-making and prioritising. It was acknowledged that this was due to participants not knowing how to properly organise their time.

*“..there was lots of little bits and pieces that actually nobody had got around to doing..probably because we didn’t know how to organise our time and prioritise it properly”* (1.3.)

#### Preparation for clinical practice

All students expressed that during their first three years of study they have had limited opportunity to develop their decision-making skills, making this aspect of clinical practice in fourth-year challenging. However, it was acknowledged that these skills are difficult to develop in a theory-based curriculum, as Years 1-3 at Dundee Medical School tends to be.

*“I think clinical decision-making is quite hard to formally teach, so we probably didn’t get that much of it from 1 ^st^ to 3 ^rd^ year”* (2.2.)

Particularly, students recognised a lack of previous teaching on the decision-making associated with patient documentation.

*“..from 1 ^st^ to 3 ^rd^ year you are never involved in the paperwork side of things”* (3.3.)

The simulated nature of the AMUWSE was discussed by all students. The authenticity of the exercise was highlighted, offering accurate insights into the clinical context. Students identified that this learning experience gave them a basis of understanding prior to their clinical rotations, ensuring they were better prepared for completing clinical tasks.

*“If you know more of what is going on in the ward, in a simulated session that is very close to real-life, then you’re more likely to be confident about joining in and actually contributing”* (4.1.)

When completing the exercise, many students perceived that there were higher expectations of them compared to what is expected on undergraduate clinical attachments. Despite these higher expectations, students could appreciate that the decision-making skills they were developing would better prepare them for life as junior doctors.

*“..although it was throwing us in the deep end, it’s something to aim for because 4 ^th^ and 5 ^th^ year, I think it’s just about training you to be an FY.. this is what you’re aiming for and these are the decisions you’re going to need to make”* (1.5.)

## Discussion

There was consensus agreement among students that the AMUWSE presented them with the reality of making clinical decisions. Instead of merely stating they would make certain decisions, as they were accustomed to, they were offered the opportunity to put these into practice - developing their critical reasoning. As this underlies the ability to make independent clinical decisions ( Benner et al., 2008), it is of vital importance undergraduates are presented with ample opportunities to develop this skill. This research also highlighted the extent to which protocols support decision-making in the clinical environment, with this seen to relieve students’ responsibility for making decisions. While it can be argued this could reduce critical reasoning, students maintained that protocols were sufficient at their stage, helping to build their confidence. Developing a sense of self-assurance in students will give them a basis for independent practice, which must be established if effective clinical decision-making is to be promoted ( Fry & MacGregor, 2014).

Clinical prioritisation continues to lack routine teaching throughout medical school, despite being acknowledged by students and graduates to be one of the key NTS required for effective patient care. It is therefore unsurprising that junior doctors consistently emphasise they do not feel adequately prepared to prioritise their workloads or time efficiently ( Brown et al., 2015). It was discovered that the AMUWSE allowed students to begin to appreciate the importance of this skill, recognising the need for prioritisation of both patients and investigations. This often came as a new experience to many, with it stressed that during the initial years of medical school there have been few formal opportunities to develop prioritisation strategies. With the recent revisions to medical teaching, students are now less likely to acquire these skills through apprenticeships and mentoring than previous generations. Therefore, there is a requirement for effective training on the subject, with research demonstrating the potential of simulated exercises to support students’ development ( McGlynn et al., 2012).

There continues to be a paucity of research supporting and directing teaching on medical handover skills, with limited evidence demonstrating transferability of these skills to the workplace ( Gordon & Findley, 2011). However, Stojan et al. (2016) have found that engaging students in frequent opportunities to practice handovers during their clinical years aids confidence and proficiency in this task. Through this, learners become more succinct and systematic in their approaches. This was consistent with the findings of this research, with students grateful for the opportunity to handover patient cases to seniors, allowing them to begin to differentiate between critical and less essential information. It was stressed the authenticity of this aspect ensured students felt better prepared for formally performing this task during their subsequent clinical rotations.

Students acknowledged that during the AMUWSE they were out of their depths with regards to the responsibilities bestowed upon them, recognising promptly when they required senior assistance. It was also identified that these interactions presented key learning opportunities for future development. This corresponds with existing research, which found following simulated exercises students gained a better understanding of when to ask for help and no longer felt afraid to do so ( McGregor et al., 2012). However, there remains a belief among junior doctors that calling for help is synonymous with incompetence, and an admission of failure ( Tallentire et al., 2012). This belief proved consistent in this study, with several students stating they were more inclined to ask colleagues for help before escalating to the consultant as they *“didn’t want to appear stupid”*, with peers perceived to offer greater personal reassurance. However, when the consultant available was a member of the regular teaching staff, students indicated they felt more comfortable seeking support. This familiarity relieved potential anxiety and ensured a more successful learning experience.

Students valued the time pressured nature of the AMUWSE, where there was a requirement for fast decision-making. It was identified this offered accurate reflections of participants’ clinical competence and of real-life practice. Nevertheless, many students stressed they did not feel adequately equipped to cope with these time constraints and thus, were unable to complete all required tasks. This perceived lack of preparedness mirrors the opinions of medical graduates across the UK, whereby they frequently describe feeling unable to efficiently managed their time, especially so in the acute care environment ( Bleakley & Brennan, 2011; Morrow et al., 2012).

All students stressed that they had had little, if any, experience of learning to make a range of clinical decisions during their initial years of medical school. This lack of preparation meant that undergraduates found the transition to clinical practice in fourth-year challenging, often feeling out of their depths. Few attempts have been reported in the literature aimed at developing clinical decision-making in the “basic science” years of medical school. However, recent research has demonstrated early encounters with ‘real’ patients in the classroom environment supports the development of these skills, aiding the eventual transition to clinical practice ( Peacock & Grande, 2015). Combining this teaching approach with exposure to simulated exercises, like the AMUWSE, could create an encompassing learning experience for students.

Additionally, the security provided by the simulated environment allowed students to develop their confidence when making independent, patient-centred decisions. This targeted training offers greater long-term benefits to undergraduates than traditional ‘on-the-job learning’, placing responsibility on the student to make a host of essential clinical decisions ( Ker et al., 2006; McGlynn et al., 2012). In addition, students could appreciate that the skills they were developing would better prepare them for life as junior doctors. While many perceived the AMUWSE to pose a greater challenge than they were familiar, this experience offered clear insights into what would be expected upon graduation regarding clinical competency.

## Limitations

This study has focused on exploring the perceptions of students regarding how the AMUWSE has influenced their clinical decision-making skills, rather than using an external objective measure to demonstrate changes in ability. However, as perceptions guide and impact on performance, confidence and ability to complete tasks ( Illing et al., 2013), this was viewed as being appropriate. Additionally, as this study was conducted in a specific institution as part of a distinct curricular component, it may not be representative of other establishments. As this exercise was merely a pilot, further research will determine the generalisability of these findings.

## Conclusions

This action research study has offered insights into the effectiveness of an AMUWSE to improve the clinical decision-making ability of medical students. This mixed methods approach has demonstrated that through participation, undergraduates begin to develop their ability to prioritise, handover information, manage time constraints and ask for help. Students were grateful for the opportunity to develop their clinical competence in a safe, simulated environment; gaining an awareness of, and preparedness for, the doctor’s decision-making responsibility.

Nevertheless, it has been identified that there remains a lack of focused clinical decision-making teaching during the initial years at Dundee Medical School. Notably, deficiencies in training on documentation and prioritisation have been emphasised during these “basic science” years. This can often lead to uncertainty for students in advance of their transition to clinical practice. To combat this issue, sufficient curriculum hours must be dedicated. While a complex process, certain interventions, such as simulation-based exercises, can prove effective in developing these skills. The relative simplicity of devising an AMUWSE ensures that any institution with an enthusiastic teaching faculty and the ability to simulate a realistic ward experience could present their students with similar learning opportunities; helping to create more competent, well-equipped junior doctors.

## Ethical Approval

Ethical approval for this study was received in December 2016 from the University of Dundee School of Medicine Research Ethics Committee (SoMREC).

## Take Home Messages

• Research continues to demonstrate both students and graduates lack effective methods for dealing with complex decisions, with many emphasising having limited experience of making clinical decisions during their undergraduate training.
• At the University of Dundee, an Acute Medical Unit ward simulation exercise (AMUWSE) has been recently incorporated into the fourth-year curriculum to offer students the opportunity to develop their procedural, examination and non-technical skills.
• An AMUWSE has been shown to improve the clinical decision-making ability of medical students, while offering invaluable insights into the nature of clinical practice.
• There remains a need for consideration of how these skills can be developed in the classroom environment during the early years of medical school.

## Notes On Contributors

Mr. Calum MacMillan is a current 4 ^th^ Year medical student who completed this research study as part fulfilment of the “Teaching in Medicine” intercalated BMSc at the University of Dundee, during the academic year 2016/2017.

Dr. George Hogg is one of the clinical skills leads at the University of Dundee School of Medicine and acted as academic supervisor throughout this study.
