# Supplementary material for: Exploring student perceptions of ward simulation as an exercise to improve decision-making skills in the clinical context
Source: MedEdPublish (2016). 2018 Jan 18;7:18. [Version 1] doi: 10.15694/mep.2018.0000018.1 (PMC10704417; doi:10.15694/mep.2018.0000018.1)
Supplement: Peer review history

# Reviewer report: Reviewer response for version 1

**DOI:** 10.21956/mep.19496.r28810

**Published:** 2018-03-27

**Authors:** Catherine Kennedy

**Affiliations:** 1 AMEE

This review has been migrated. The reviewer awarded 3 stars out of 5 This is an interesting article that highlights the gap between theory and practice in medical education, and discusses the perceived impact of an important intervention to introduce medical students to clinical decision making. The research outcomes are clearly related to the research literature, and the discussion highlights the key findings alongside suggestions for future development. There are a few areas in which further details and elaboration would have strengthened the article: • It would have been interesting to learn more about AMUWSE, both to provide context to this research, and for sharing examples of practice with the medical education community; • The survey had a very low response rate (13/168) and it would be interesting to know what proportion of the 168 had completed the training (the intended sample) and to reflect on whether a different approach could have been used to increase the response rate.• In Table 1 it would be useful to know the grading behind the mean scores (e.g. was it 1=Strongly Disagree – 5= Strongly Agree?)• It would be helpful to know more about the questions asked in the qualitative part of this research, for example by including your interview guide as an appendix.

Reviewer Expertise:

NA

No decision status is available

# Reviewer report: Reviewer response for version 1

**DOI:** 10.21956/mep.19496.r28809

**Published:** 2018-01-20

**Authors:** Tripti Srivastava

**Affiliations:** 1 Datta Meghe Institute of Medical Sciences Deemed University

This review has been migrated. The reviewer awarded 5 stars out of 5 Clinical decision making, a vital competence of a health professional is dealt suitably in the present a study by piloting an Acute Medical Unit ward Simulation Exercise (AMUWSE) for fourth year medical students. The learners were given ‘on job training’ by providing opportunity to practice procedural, examination and non technical skills. The manuscript depicts the quantitative and qualitative outcomes of the intervention, which is encouraging. The themes emerging out of the group interviews viz. making clinical decisions, prioritising, handing over information, asking for help, dealing with time constraints and preparation for clinical practice are adequately elucidated. It is an action research well articulated ,executed and presented. It will be interesting if the authors give further insights about AMUWSE so as to replicate in other medical schools. Congratulations for the commendable job. Much recommended!

Reviewer Expertise:

NA

No decision status is available
